# Rosetting revisited: a critical look at the evidence for host erythrocyte receptors in *Plasmodium falciparum* rosetting

**DOI:** 10.1017/S0031182019001288

**Published:** 2020-01

**Authors:** Fiona McQuaid, J. Alexandra Rowe

**Affiliations:** Institute of Immunology and Infection Research, Centre for Immunity, Infection and Evolution, School of Biological Sciences, Ashworth Laboratories, Kings Buildings, Charlotte Auerbach Rd, Edinburgh, EH9 3FL, UK

**Keywords:** ABO blood group, adjunctive therapy, cell adhesion, *Plasmodium*, receptors, severe malaria pathogenesis

## Abstract

Malaria remains a major cause of mortality in African children, with no adjunctive treatments currently available to ameliorate the severe clinical forms of the disease. Rosetting, the adhesion of infected erythrocytes (IEs) to uninfected erythrocytes, is a parasite phenotype strongly associated with severe malaria, and hence is a potential therapeutic target. However, the molecular mechanisms of rosetting are complex and involve multiple distinct receptor–ligand interactions, with some similarities to the diverse pathways involved in *P. falciparum* erythrocyte invasion. This review summarizes the current understanding of the molecular interactions that lead to rosette formation, with a particular focus on host uninfected erythrocyte receptors including the A and B blood group trisaccharides, complement receptor one, heparan sulphate, glycophorin A and glycophorin C. There is strong evidence supporting blood group A trisaccharides as rosetting receptors, but evidence for other molecules is incomplete and requires further study. It is likely that additional host erythrocyte rosetting receptors remain to be discovered. A rosette-disrupting low anti-coagulant heparin derivative is being investigated as an adjunctive therapy for severe malaria, and further research into the receptor–ligand interactions underlying rosetting may reveal additional therapeutic approaches to reduce the unacceptably high mortality rate of severe malaria.

## Introduction

**Fig. 1.**
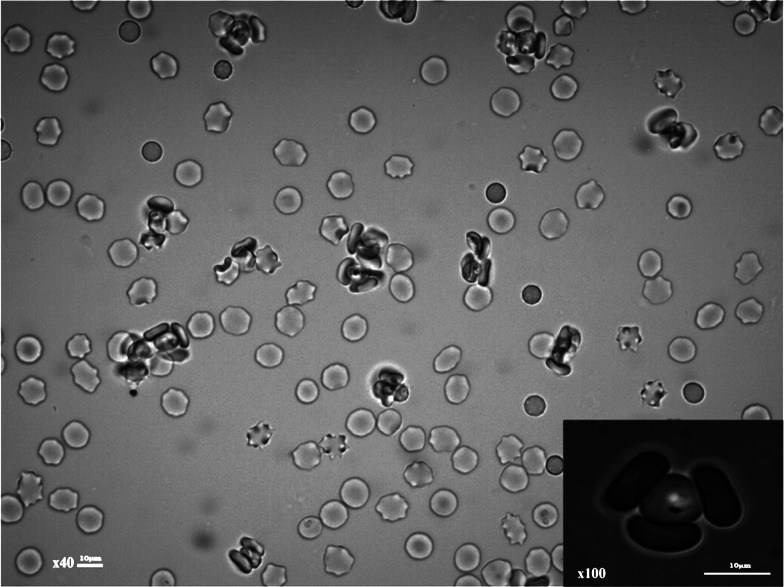
*Plasmodium falciparum* rosetting in an *in vitro* culture. Rosettes consisting of clusters of infected and uninfected erythrocytes are shown. Inset image shows a single infected erythrocyte (centre) and three adherent uninfected erythrocytes. Images were taken using a Yenway microscope camera on a Leica DM LB2 fluorescent microscope using the ×40 and ×100 (inset) objectives.

Rosetting is a *Plasmodium falciparum* infected erythrocyte (IE) adhesion phenotype that is associated with severe malaria in sub-Saharan Africa (summarized in Doumbo *et al*., 2009). It is a form of cell adhesion in which erythrocytes infected with mature, asexual parasites bind to uninfected erythrocytes to form clusters of cells (Fig. 1). Rosetting is a phenotypically variable property, which is common in parasite isolates collected from severe malaria patients, but infrequent in parasites from uncomplicated malaria cases. For culture-adapted *P. falciparum* isolates, only a subset of parasite lines can be selected *in vitro* for the rosetting phenotype, and many of the commonly used laboratory strains such as 3D7, rosette poorly or not at all. The relative rarity of rosetting in culture-adapted parasite lines may explain why rosetting is studied infrequently, despite being a virulence-associated phenotype in clinical isolates.

Rosetting can contribute to IE sequestration and microvascular congestion, leading to obstruction to blood flow (Kaul *et al*., 1991), one of the major pathological events in severe falciparum malaria contributing to inflammation, tissue damage and organ failure (Miller *et al*., 2002; White *et al*., 2013). Rosetting also causes membrane changes in uninfected erythrocytes that may contribute to phagocytic removal and anaemia (Uyoga *et al*., 2012). In Africa, high levels of rosetting occur in parasites sampled from severe malaria patients with all clinical types of disease including cerebral malaria (Carlson *et al*., 1990; Treutiger *et al*., 1992; Ringwald *et al*., 1993; Rowe *et al*., 1995; Kun *et al*., 1998; Doumbo *et al*., 2009), severe malarial anaemia (Newbold *et al*., 1997; Doumbo *et al*., 2009) and respiratory distress (Warimwe *et al*., 2012). Rosette-like clusters of cells have been seen in the microvasculature in histological studies of fatal malaria cases (Dondorp *et al*., 2004; Barrera *et al*., 2018). The major *Plasmodium* species that infect humans are all able to form rosettes (Udomsanpetch *et al*., 1995; Angus *et al*., 1996; Chotivanich *et al*., 1998; Lowe *et al*., 1998). However, the link between severity of disease and rosetting is confined to *P. falciparum*, possibly due to the unique ability to bind both endothelial cells and uninfected erythrocytes simultaneously (Udomsangpetch *et al*., 1992; Adams *et al*., 2014), such that *P. falciparum* rosetting IEs are sequestered and are not seen in peripheral blood. Recently it has been suggested that rosetting may contribute to anaemia in *Plasmodium vivax* infections (Marín-Menéndez *et al*., 2013).

The biological function of rosetting *in vivo* remains unknown. Rosettes may shield IEs from host immune attack, or close contact with uninfected erythrocytes in rosettes might enhance merozoite invasion (Wahlgren *et al*., 1989; Deans and Rowe, 2006). However, firm evidence to support either of these hypotheses is lacking. Most rosetting parasite isolates form larger, stronger rosettes with blood group A erythrocytes compared to other blood groups (Carlson and Wahlgren, 1992), and these group A rosettes may shield IEs to reduce antibody binding to parasite variant surface antigens (VSAs) (Moll *et al*., 2015). Whether this translates into the reduced clearance of IEs and subsequent higher parasite burdens *in vivo* is unclear, although some studies have noted a positive correlation between rosetting and parasitaemia (Rowe *et al*., 2002). Another study showed that rosetting does not prevent IgG-mediated phagocytosis of IEs (Stevenson *et al*., 2015*a*), although experiments were only performed in group O cells. Parasite invasion of erythrocytes is not increased *in vitro* in rosetting compared to isogenic non-rosetting parasites (Clough *et al*., 1998; Deans and Rowe, 2006; Ribacke *et al*., 2013), nor in the presence of larger rosettes (Moll *et al*., 2015). However, *in vivo* studies using splenectomized *Saimiri sciureus* monkeys demonstrated a 1.5 times higher parasite multiplication rate with rosetting compared to isogenic non-rosetting parasites (Le Scanf *et al*., 2008). This suggests either increased invasion or decreased clearance of rosetting parasites *in vivo*, which requires further investigation.

**Fig. 2.**
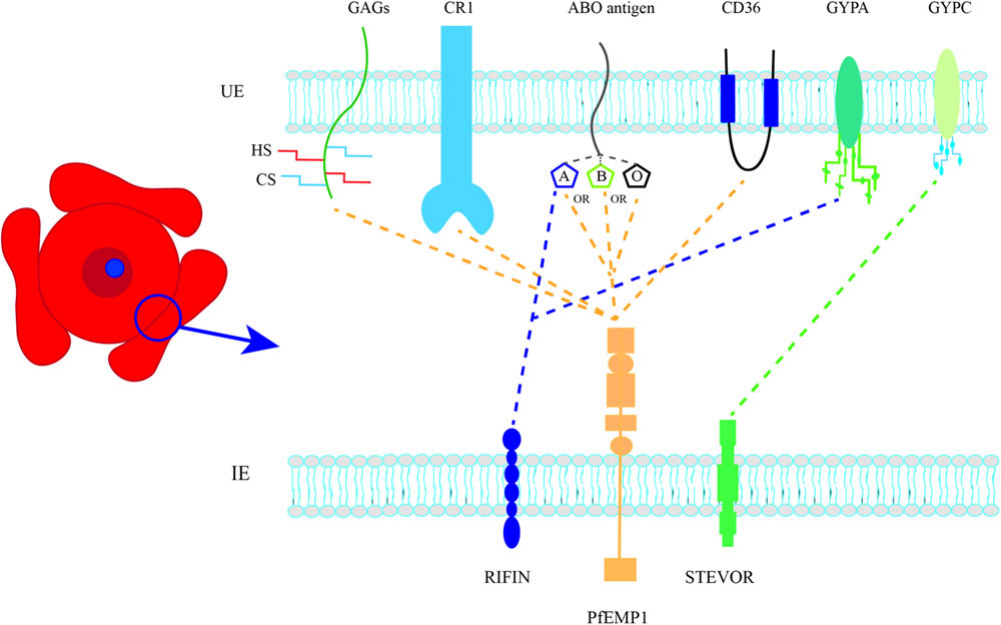
Parasite-derived adhesion ligands and host receptors that interact to form rosettes. UE, uninfected erythrocyte; IE, infected erythrocyte; GAGs, glycosaminoglycans; HS, heparan sulphate; CS, chondroitin sulphate; CR1, complement receptor 1; GYPA, glycophorin A; GYPC, glycophorin C. Dotted lines represent proposed host receptors for each parasite ligand.

This review will discuss the molecular mechanisms of rosetting and describe recent advances exploring the potential of rosetting as a therapeutic target in severe *P. falciparum* malaria. Rosetting is a complex cell adhesion phenotype involving parasite adhesion molecules on the IE surface and host receptors on uninfected erythrocytes (Fig. 2). Current evidence suggests that there are multiple distinct pathways of rosette formation, similar to the diverse pathways involved in merozoite invasion of erythrocytes (Cowman *et al*., 2017). Interestingly, although the parasite molecules that mediate rosetting are different from those involved in merozoite invasion, both sets of proteins have ‘Duffy-Binding-Like’ adhesion domains and many of the same host erythrocyte receptors are used (e.g. glycophorin A, glycophorin C and complement receptor one). The diversity in *P. falciparum* merozoite invasion pathways is thought to have evolved to allow parasites to successfully establish infections despite host genetic variation and/or development of host antibodies blocking single pathways. The same arguments can be applied to rosetting, and the existence of multiple rosetting pathways suggests that there has been significant selection pressure in favour of the phenotype, and that rosetting somehow improves parasite fitness.

Several recent reviews have discussed the parasite adhesion molecules involved in rosetting (Hviid and Jensen, 2015; Wang and Hviid, 2015; Yam *et al*., 2017), so these will not be described in detail here. Briefly, multiple studies have identified members of the VSA family *P. falciparum* erythrocyte membrane protein one (PfEMP1) as rosette-mediating adhesion molecules (Rowe *et al*., 1997; Vigan-Womas *et al*., 2008, 2011; Albrecht *et al*., 2011; Ghumra *et al*., 2012), and recent reports suggest that other VSAs such as RIFIN (Goel *et al*., 2015) and STEVOR (Niang *et al*., 2014) may also contribute to rosette formation. Further work is needed to determine the relative contributions of the different VSAs to rosetting, especially in clinical isolates.

Host serum proteins such as IgM, *α*2macroglobulin, albumin and fibrinogen also contribute to rosetting, either by binding directly to parasite adhesion molecules or by non-specific erythrocyte aggregating effects (Scholander *et al*., 1996; Treutiger *et al*., 1999; Luginbuhl *et al*., 2007; Ghumra *et al*., 2008, 2012; Semblat *et al*., 2015; Stevenson *et al*., 2015*a*, 2015*b*). The extent to which host serum proteins influence rosetting, sequestration and microvascular obstruction *in vivo* is unknown, and would be a valuable area of future study.

## Rosetting receptors on host erythrocytes

A number of different molecules on uninfected erythrocytes have been proposed as receptors for *P. falciparum* rosetting (Fig. 2 and Table 1), and multiple receptor–ligand interactions may contribute to rosetting in any given parasite isolate. Some of the proposed rosetting receptor molecules, including blood group A and B sugars, heparan sulphate (HS)-like molecules and complement receptor one (CR1) are widely accepted as having a role in rosetting, whereas other recent candidates such glycophorin A (GYPA) and glycophorin C (GYPC) are less well-authenticated. However, a close examination of the underlying data shows that in most cases, the evidence is incomplete, as discussed in detail below.

**Table 1.** Summary of host erythrocyte receptors for *Plasmodium falciparum* rosetting

Name	Characteristics	Studies^a^	Comments
ABO blood group antigens	Differ based on terminal sugar: A = *N*-acetyl-D-galactosamine, B = D-galactose, O (H-antigen) = L-Fucose O is a predominant blood group in sub-Saharan Africa Blood group O protects against severe malaria (Rowe *et al*., 2007; Fry *et al*., 2008; Tekeste and Petros, 2010; Rout *et al*., 2012; Malaria Genomic Epidemiology Network, 2014; Ndila *et al*., 2018; Degarege *et al*., 2019)	Larger rosettes in parasites cultured in A, B, AB compared to O (Carlson and Wahlgren, 1992; Udomsangpetch *et al*., 1993; Barragan *et al*., 2000*b*) Parasites from group O patients have lower mean rosette frequencies than those from non-O patients (Rowe *et al*., 1995; Rowe *et al*., 2007; Rout *et al*., 2012) Rosettes from group O patients are more easily disrupted by immune sera and removal of A/B antigen decreases rosette size (Barragan *et al*., 2000*b*) Blood group antigen binding site mapped to NTS-DBL*α* the domain of PfEMP1-VarO (Vigan-Womas *et al*., 2012)	Blood group A antigen is the most well-validated host rosetting receptor Both PfEMP1 (Vigan-Womas *et al*., 2012) and RIFINs (Goel *et al*., 2015) may interact with A antigen Challenging to manipulate therapeutically
Complement receptor 1 (CR1)	Membrane glycoprotein responsible for regulating the complement system (Thielen *et al*., 2018) Polymorphisms affect CR1 copy number, molecular weight and sequence (Schmidt *et al*., 2015) RBC CR1 deficiency protects in medium-high (Cockburn *et al*., 2004; Sinha *et al*., 2009; Rout *et al*., 2011; Panda *et al*., 2012) but not low malaria transmission areas (Nagayasu *et al*., 2001; Teeranaipong *et al*., 2008). CR1 Knops blood group polymorphisms associated with severe malaria (Opi *et al*., 2018)	Rosetting reduced in CR1 deficient erythrocytes (Rowe *et al*., 1997) Soluble CR1 and CR1 antibodies disrupt rosettes in some parasite isolates (Rowe *et al*., 1997; Rowe *et al*., 2000; Vigan-Womas *et al*., 2012) Essential region mapped to the C3b binding site on CR1 (Rowe *et al*., 2000)	Further work needed to assess the relative importance of CR1 in rosetting isolates and potential as a therapeutic target Soluble recombinant CR1 has been considered for therapeutic use in humans, e.g. cardiac and renal disease (Li *et al*., 2006; Reddy *et al*., 2017)
Heparan sulphate (HS)^b^	Glycosaminoglycan Heparin is a highly sulfated form of HS that is only found in mast cells HS is a receptor for *P. falciparum* sporozoite invasion of hepatocytes (Frevert *et al*., 1993) HS is a receptor for infected erythrocyte cytoadherence to endothelial cells (Vogt *et al*., 2003; Adams *et al*., 2014)	Heparin partially disrupts rosettes in some isolates (Udomsangpetch *et al*., 1989; Carlson *et al*., 1992; Rogerson *et al*., 1994; Rowe *et al*., 1994; Barragan *et al*., 1999) Heparinase treatment reported to reduce rosetting in two culture-adapted parasite lines (Barragan *et al*., 1999) Heparin binds to rosetting IE (Barragan *et al*., 2000*a*; Heddini *et al*., 2001) and to rosette-mediating PfEMP1 (Barragan *et al*., 2000*a*; Vogt *et al*., 2003; Juillerat *et al*., 2010; Juillerat *et al*., 2011; Adams *et al*., 2014)	Limited evidence that HS is present on mature RBCs (Vogt *et al*., 2004) Further work needed to determine whether HS is present on normal erythrocytes and acts as a rosetting receptor Therapeutic potential due to PfEMP1 binding and rosette disruption. Clinical trials of low anticoagulant heparin ongoing (Leitgeb *et al*., 2017)
Chondroitin sulphate (CS)	Glycosaminoglycan Receptor for infected erythrocyte placental sequestration in pregnancy malaria (Fried and Duffy, 1996)	Soluble CS did not disrupt rosettes (Rogerson *et al*., 1994; Rowe *et al*., 1994) Chondroitinase treatment reduced rosetting in one parasite line only (Barragan *et al*., 1999)	No evidence that CS is present on mature RBC Minimal evidence for a role in rosetting
CD36	Widely distributed membrane protein and scavenger receptor (Silverstein and Febbraio, 2009) Deficiency is common in Africa but not associated with severe malaria (Fry *et al*., 2009)	Antibodies disrupt rosettes in single culture-adapted line only (Handunnetti *et al*., 1992) PfEMP1 variants that mediate rosetting are group A types that do not bind CD36 (Robinson *et al*., 2003)	Minimal evidence for a widespread role in rosetting
Glycophorin C (GYPC)	Red cell membrane protein responsible for Gerbich blood group (Jaskiewicz *et al*., 2018) Receptor for merozoite invasion of erythrocytes (Maier *et al*., 2003) ‘Gerbich-negative’ blood group common in Melanesians (Patel *et al*., 2001), but no evidence yet for association with protection against severe malaria	Reduced rosetting with GYPC antibodies and GYPC knockdown RBCs (Niang *et al*., 2014) (single culture-adapted parasite line tested) Gerbich-negative erythrocytes formed rosettes normally with five *P. falciparum* lines (Rowe *et al*., 1997) Possible role in *P. vivax* rosetting (Lee *et al*., 2014)	Further work needed to assess the relative importance of GYPC in *P. falciparum* rosetting isolates and potential as a therapeutic target
Glycophorin A (GYPA)	Sialoglycoprotein which, along with glycophorin B, constitutes the MNS blood group Receptor for merozoite invasion of erythrocytes (Sim *et al*., 1994) GYPA polymorphisms are associated with protection against severe malaria (Band *et al*., 2015; Leffler *et al*., 2017).	GYPA-deficient erythrocytes showed reduced rosetting with RIFIN transfected parasites (Goel *et al*., 2015) GYPA antibodies had no inhibitory effect on rosetting (Lee *et al*., 2014) (Niang *et al*., 2014) GYPA null erythrocytes formed rosettes with five culture-adapted *P. falciparum* lines (Rowe *et al*., 1997)	Further work needed to assess the relative importance of GYPA in *P. falciparum* rosetting isolates and potential as a therapeutic target
Unknown receptor/s	Possibly carbohydrate or protease-resistant protein	Protease and heparinase treated erythrocytes capable of forming rosettes (Udomsangpetch *et al*., 1989; Rowe *et al*., 1994)	Further work needed to identify novel rosetting receptors

a Parasite strains used are not consistent between studies with a wide range of culture-adapted and clinical isolates in use. Results are therefore not necessarily generalizable from single studies.

b Many studies included here use heparin instead of/in addition to heparan sulphate.

### Evidence needed to establish a role for a specific host receptor in rosetting

In order to prove that a particular molecule acts as a host receptor for *P. falciparum* rosetting, a variety of different types of evidence have been provided. Essential data include proof that the molecule in question is found on normal human erythrocytes and that erythrocytes lacking the molecule show reduced/absent rosetting. Direct binding between IEs and/or recombinant parasite adhesion proteins and the receptor molecule should be demonstrated. Ideally, a crystal structure of the parasite adhesion molecule–host receptor complex should show the precise binding interaction site. Supportive evidence includes the ability of antibodies against the receptor or soluble receptor proteins to inhibit rosetting, and biochemical approaches to remove or alter the receptor on erythrocytes. Human genetic evidence can also provide indirect supportive evidence that particular molecules are important in life-threatening malaria. Several putative rosetting receptors have high-frequency polymorphisms in populations from malaria endemic regions that reduce rosetting and are associated with protection against severe malaria and death [reviewed in Rowe *et al*. (2009*a*, 2009*b*)]. These various lines of evidence are summarized below for each potential host rosetting receptor.

### Blood group A and B trisaccharides

**Fig. 3.**
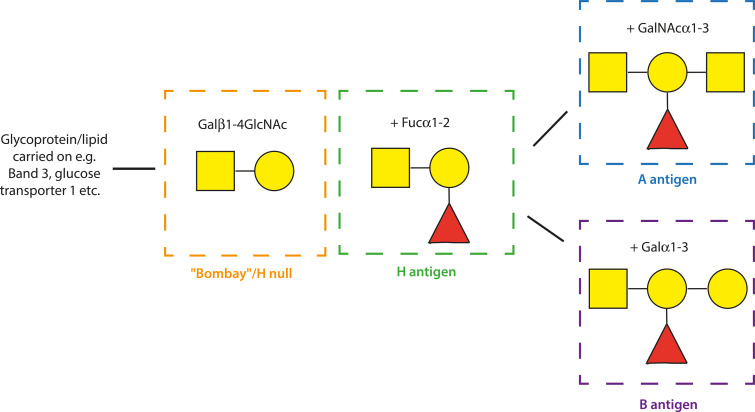
Diagram of the ABO blood group sugars. Schematic representation of the terminal structure of the A (blue square), B (purple) H (green; H is the antigen carried on blood group O erythrocytes) and Bombay (orange) antigens. Yellow circle: D-Galactose (Gal), yellow square: *N*-acetyl-D-galactosamine (GalNac), red triangle: L-Fucose (Fuc). The symbols *α* and *β* indicate the position of the hydroxyl group and the numbers indicate the specific carbon atoms that are linked between the sugars. The H, A and B antigens are synthesized by a series of glycosyltransferase enzymes that add monosaccharides to create oligosaccharide chains attached to lipids and proteins in the erythrocyte membrane.

The most well-validated rosetting receptors are the blood group A and B trisaccharides (Fig. 3). *In vitro* experiments have shown that rosetting parasites have a ‘preference’ for blood groups A, B or AB rather than O (Carlson and Wahlgren, 1992; Udomsangpetch *et al*., 1993; Barragan *et al*., 2000*b*; Pipitaporn *et al*., 2000; Vigan-Womas *et al*., 2012; Moll *et al*., 2015). This varies by parasite genotype, with A-preference being the commonest. Clinical isolates from non-O (i.e. A, B or AB) patients show higher levels of rosetting than isolates from group O patients in studies from sub-Saharan Africa (Rowe *et al*., 1995, 2007) and India (Rout *et al*., 2012), although the same result was not seen in one Thai study (Lee *et al*., 2014). When parasites are cultured in their ‘preferred’ blood group, they form larger, stronger rosettes that are more resistant to disruption by antibodies or chemical agents than in group O cells (Carlson and Wahlgren, 1992; Barragan *et al*., 2000*b*; Ch'ng *et al*., 2016). Enzymatic removal of the terminal sugars (*N*-acetyl-D-galactosamine for A and D-galactose for B) results in smaller, weaker rosettes, equivalent to those seen in group O erythrocytes (Barragan *et al*., 2000*b*). Rosettes do, however, still occur with blood group O erythrocytes (that express the H antigen), and also in Bombay phenotype red cells that lack the ABO blood group core fucose residue (Fig. 3) (Carlson and Wahlgren, 1992; Rowe *et al*., 1997). This indicates that other red cell surface molecules in addition to the A and B antigens can act as host receptors for rosette formation.

For the blood group A-preferring parasite line, Palo Alto 89F5, direct binding between the VarO PfEMP1 adhesion molecule and the blood group A trisaccharide was shown by Surface Plasmon Resonance (Vigan-Womas *et al*., 2012). The VarO PfEMP1 variant also binds to the B trisaccharide, but with lower affinity (Vigan-Womas *et al*., 2012). A crystal structure of the PfEMP1 N-terminal region was obtained and the A-trisaccharide binding site mapped (Vigan-Womas *et al*., 2012). A recent study suggests that *P. falciparum* RIFIN molecules may also be able to interact with blood group A sugars to contribute to rosette formation (Goel *et al*., 2015), although direct RIFIN-A trisaccharide interaction was not shown.

The importance of the A and B antigens in rosetting is emphasized by the fact that the non-O blood groups are associated with increased risk of severe malaria and death compared to O (Rowe *et al*., 2007; Fry *et al*., 2008; Tekeste and Petros, 2010; Rout *et al*., 2012; Malaria Genomic Epidemiology Network, 2014; Ndila *et al*., 2018; Degarege *et al*., 2019). Reduced rosetting in blood group O, and therefore reduced microvascular obstruction and reduced downstream pathological effects, is the proposed mechanism for the protective association with group O (Udomsangpetch *et al*., 1993; Rowe *et al*., 2007). ABO blood group does not influence parasite burden (Rowe *et al*., 2007; Degarege *et al*., 2019), and evidence for an effect of ABO on *P. falciparum* invasion or other host–parasite interactions is conflicting and requires further study (Chung *et al*., 2005; Wolofsky *et al*., 2012; Pathak *et al*., 2016; Theron *et al*., 2018). The ABH antigens are known to be present on endothelial cells (Ito *et al*., 1990) and it is likely, but has not been shown experimentally, that cytoadhesion and overall levels of sequestration of rosetting parasites are enhanced in group A/B/AB patients compared to O.

Despite the progress in identifying the A and B trisaccharides as rosetting receptors and key genetic determinants of host susceptibility to severe malaria, there have been no attempts to develop specific therapies to block *P. falciparum* interaction with A/B antigens. The PfEMP1-blood group A trisaccharide binding pair described above (Vigan-Womas *et al*., 2012) remains the most clearly defined molecular interaction between parasite ligand and host receptor in rosetting, and could be used as a starting point to develop rosette-blocking therapeutics. Vigan-Womas *et al.* did report that the interaction between PfEMP1 and the A and B trisaccharides is indirectly inhibited by heparin (Vigan-Womas *et al*., 2012), and the development of a heparin-derivative as a potential adjunctive therapy for severe malaria is described below (Leitgeb *et al*., 2017).

### Complement receptor one (CR1, CD35)

CR1 is a red cell membrane glycoprotein that regulates complement activation on cell surfaces (Thielen *et al*., 2018) and carries the Knops Blood Group antigens (Moulds, 2010). In malaria, CR1 plays a role in both rosetting and parasite invasion of erythrocytes (Schmidt *et al*., 2015). CR1 was first identified as a rosetting receptor from a screen of 23 naturally occurring erythrocyte null mutants, each missing a particular blood group molecule or membrane glycoprotein (Rowe *et al*., 1997). The only variant to show substantially reduced rosetting with five *P. falciparum* parasite lines was Knops null cells, which are deficient in CR1. Normally, erythrocytes have between 100 and 1000 molecules of CR1 per cell (Wilson *et al*., 1986), whereas Knops null cells have fewer than 100 molecules per cell (Moulds *et al*., 1992). Erythrocytes with fewer than 50 CR1 molecules per cell form rosettes poorly (Rowe *et al*., 1997), with normal rosetting occurring above a threshold of around 100 molecules per cell (JA Rowe, unpublished data).

Soluble CR1 and CR1 antibodies were shown to inhibit rosetting in some but, not all *P. falciparum* rosetting laboratory strains and clinical isolates, with only monoclonal antibodies (mAbs) that map to the C3b binding site on CR1 being effective inhibitors (Rowe *et al*., 1997, 2000; Vigan-Womas *et al*., 2012). A recent paper suggested that the commercially available CR1 mAb E11 that recognizes epitopes outside the C3b binding site (Nickells *et al*., 1998) may inhibit *P. falciparum* rosetting (Lee *et al*., 2014), but this was not seen in our hands (Rowe *et al*., 2000). Further evidence of a role for CR1 in rosetting came from the expression of recombinant PfEMP1 domains in COS-7 cells, which bound to normal erythrocytes but not to CR1-deficient cells (Rowe *et al*., 1997).

Despite these supportive data, direct binding of IEs to CR1 protein has not been demonstrated, and recombinant rosette-mediating PfEMP1 proteins produced in *E. coli* (Ghumra *et al*., 2012) do not bind to CR1 in Surface Plasmon Resonance experiments (Tetteh-Quarcoo *et al*., 2012). This could reflect a genuine lack of interaction between the two molecules, or could be due to technical reasons (e.g. the recombinant CR1 used in experiments was produced in mouse rather than human cells, whereas CR1 glycosylation, which may affect function, is cell-type specific) (Lublin *et al*., 1986). It is also possible that a serum protein mediates the interaction between PfEMP1 on IEs and CR1 on uninfected erythrocytes, as the original experiments were all performed in the presence of serum (Rowe *et al*., 1997).

Human genetic studies provide additional support for the importance of CR1 in malaria host–parasite interactions. Erythrocyte CR1 deficiency is common in some malaria-endemic countries such as Papua New Guinea (Cockburn *et al*., 2004) and India (Sinha *et al*., 2009), and is associated with protection against severe malaria in medium to high transmission areas (Cockburn *et al*., 2004; Sinha *et al*., 2009; Rout *et al*., 2011; Panda *et al*., 2012). However, erythrocyte CR1 deficiency may be detrimental in areas such as Thailand, where malaria transmission is low (Nagayasu *et al*., 2001; Teeranaipong *et al*., 2008). There is also evidence that the CR1 Swain Langley 2 (Sl2) Knops blood group polymorphism that is common in African populations (Moulds, 2010) is associated with protection against severe malaria (Thathy *et al*., 2005; Opi *et al*., 2018). Red cells carrying the Sl2 antigen on CR1 show reduced rosetting (Rowe *et al*., 1997; Opi *et al*., 2018), and Sl2 may have additional effects on complement activation and regulation (Opi *et al*., 2018).

Overall, the ability of CR1 mAbs and soluble protein to reverse rosettes suggests that CR1 plays a role in rosetting for some *P. falciparum* isolates. However, further work is needed to fully investigate the molecular interactions between parasite adhesion molecules and CR1, and to explore the potential for CR1 reagents (Li *et al*., 2006; Reddy *et al*., 2017) as therapeutic disruptors of rosetting.

### Heparan sulphate and chondroitin sulphate

The glycosaminoglycans HS and chondroitin sulphate (CS) are found on cell surfaces and in the extracellular matrix of many tissues, and have a role in multiple aspects of the *P. falciparum* life cycle including hepatocyte invasion (Frevert *et al*., 1993), endothelial cell cytoadherence (Vogt *et al*., 2003; Adams *et al*., 2014) and, for CS, placental sequestration (Fried and Duffy, 1996). A number of papers have showed that heparin (which is a highly-sulphated form of HS found only in mast cells) can partially disrupt rosettes in about one-third to one-half of *P. falciparum* clinical isolates *in vitro* (Udomsangpetch *et al*., 1989; Carlson *et al*., 1992; Rogerson *et al*., 1994; Rowe *et al*., 1994; Barragan *et al*., 1999). It was shown that treating erythrocytes with heparinase III, which selectively cleaves HS chains, reduces rosetting in two *P. falciparum* lines (Barragan *et al*., 1999), and therefore suggested that ‘HS-like’ molecules on red cells are receptors for rosetting (Chen *et al*., 2000). However, there has been only one paper reporting the existence of HS on normal human erythrocytes (Vogt *et al*., 2004) and we have been unable to confirm this, and unable to detect any rosette-reducing effect of heparinase III in a range of parasite lines (McQuaid and Rowe, unpublished data).

Fluorescently-labelled heparin does bind to the surface of erythrocytes infected with rosetting parasites more than non-rosetting lines (Barragan *et al*., 2000*a*; Heddini *et al*., 2001), and some rosette-mediating PfEMP1 variants bind directly to heparin (Barragan *et al*., 2000*a*; Vogt *et al*., 2003; Juillerat *et al*., 2010, 2011; Adams *et al*., 2014). The heparin binding site in the N-terminal region of the varO PfEMP1 variant was mapped onto a crystal structure (Juillerat *et al*., 2011), and shown to be on the opposite side of the molecule from the erythrocyte binding site (Vigan-Womas *et al*., 2012). Hence, the rosette-disrupting effect of heparin is not due to direct blocking of receptor binding, but may result from aggregating PfEMP1 monomers and preventing their interaction with erythrocyte receptors (Vigan-Womas *et al*., 2012). Similarly, for another rosette-mediating PfEMP1 variant IT4var60, site-directed mutagenesis studies of recombinant proteins showed that mutations that disrupt heparin binding are distinct from mutations that disrupt erythrocyte binding, indicating that heparin-like molecules are not the main host rosetting receptor in this case (Angeletti *et al*., 2015).

Overall, whether HS is present on normal erythrocytes and is a host receptor for rosetting requires further confirmation. HS in present on the luminal surface of microvascular endothelial cells (albeit at a much lower density than on basolateral surfaces) (de Agostini *et al*., 1990; Stoler-Barak *et al*., 2014), therefore interactions between IE and endothelial HS (Vogt *et al*., 2003; Adams *et al*., 2014) are physiologically relevant and are likely to contribute to cytoadherence and sequestration *in vivo*.

Despite the uncertainty on the precise role of HS as an erythrocyte rosetting receptor, heparin and other sulphated glycoconjugate compounds have clear potential as adjunctive therapies for severe malaria due to their rosette-disrupting effects (Udomsangpetch *et al*., 1989; Carlson *et al*., 1992; Rogerson *et al*., 1994; Rowe *et al*., 1994; Kyriacou *et al*., 2007). There are reports of successful heparin treatment in severe malaria (Rampengan, 1991) but its use is not recommended due to a high incidence of bleeding complications (World Health Organisation, 1986). As an alternative, Wahlgren and coworkers have developed a low anti-coagulant heparin derivative, Sevuparin, that reverses rosetting and cytoadherence in some *P. falciparum* isolates (Leitgeb *et al*., 2011; Saiwaew *et al*., 2017) and also blocks merozoite invasion (Leitgeb *et al*., 2017). Sevuparin has been shown to be safe in adults with uncomplicated malaria (Leitgeb *et al*., 2017), but has not yet been tested in severe malaria patients.

The evidence for CS as a rosetting receptor is minimal. One report shows that rosetting in the *P. falciparum* line TM284 was partially inhibited by soluble CS and by chondroitinase enzyme treatment of erythrocytes, and that several clinical isolates showed reduced rosetting in the presence of CS (Barragan *et al*., 1999). However, other studies have found no effect of CS on rosetting in a variety of culture-adapted lines and clinical isolates (Rogerson *et al*., 1994; Rowe *et al*., 1994). There is also no convincing evidence that CS is found on the surface of normal human erythrocytes. Overall, current data do not support a role for CS in rosetting.

### CD36

The membrane glycoprotein CD36 is a scavenger receptor for oxidized lipoproteins and a fatty acid translocase (Silverstein and Febbraio, 2009). It is expressed on a variety of cell types including monocytes, macrophages, platelets, microvascular endothelial cells and adipocytes (Silverstein and Febbraio, 2009), and at low levels on erythrocytes (van Schravendijk *et al*., 1992). The binding of PfEMP1 (group B and C variants) to CD36 on microvascular endothelial cells plays a major role in *P. falciparum* sequestration (Baruch *et al*., 1996; Robinson *et al*., 2003). Almost all *P. falciparum* isolates bind to CD36, and increased CD36 binding (Newbold *et al*., 1997; Ochola *et al*., 2011) and predominant expression of group B and C PfEMP1 (Kraemer and Smith, 2006; Kyriacou *et al*., 2006) are associated with uncomplicated malaria.

The role of CD36 in rosetting is less clear. Anti-CD36 mAbs are capable of disrupting rosettes in a single culture-adapted parasite line, Malayan Camp (Handunnetti *et al*., 1992), but not in a wide range of other laboratory lines or clinical isolates (Udomsangpetch *et al*., 1989; Wahlgren *et al*., 1992; Rowe *et al*., 2000; Niang *et al*., 2014). The PfEMP1 variants identified as parasite rosetting ligands (Rowe *et al*., 1997; Vigan-Womas *et al*., 2011; Ghumra *et al*., 2012) are mostly of the group A type, which do not bind to CD36 (Robinson *et al*., 2003).

Intriguingly, while CD36 deficiency is fairly common in African populations, large-scale genetic studies have shown that CD36 polymorphisms do not influence severe malaria risk (Fry *et al*., 2009). There is some evidence that interaction between IEs and CD36 may benefit the host, as CD36 may contribute to innate immune clearance of IEs and platelet-mediated parasite death (McGilvray *et al*., 2000; McMorran *et al*., 2012; Cabrera *et al*., 2014). Overall, it is unlikely that CD36 is a clinically significant rosetting receptor or a useful therapeutic target in severe malaria (Cabrera *et al*., 2014).

### Glycophorin C (GYPC; GPC; CD236)

GYPC is a red cell membrane glycoprotein that carries the Gerbich blood group antigens (Jaskiewicz *et al*., 2018). It is a *P. falciparum* invasion receptor bound by the merozoite protein EBA-140/BAEBL (Maier *et al*., 2003; Mayer *et al*., 2006). Recently, two studies have suggested that GYPC is a rosetting receptor for both *P. falciparum* (Niang *et al*., 2014) and *P. vivax* (Lee *et al*., 2014; Niang *et al*., 2014). Niang *et al.* showed that the rosetting of a 3D7-derived *P. falciparum* laboratory strain (5A-R+) was partially inhibited by a GYPC mAb (clone Ret40f) and by soluble recombinant GYPC (Niang *et al*., 2014). Furthermore, cultured GYPC knockdown erythrocytes failed to rosette, providing strong evidence that GYPC is an essential rosetting receptor for 5A-R+ parasites (Niang *et al*., 2014). Other parasite lines or clinical isolates were not tested, therefore the wider role of GYPC in *P. falciparum* rosetting was not determined.

Lee *et al*. (2014) focussed mainly on *P. vivax*, but also assessed the ability of GYPC mAb fragments to inhibit rosette formation in ten *P. falciparum* clinical isolates from Thailand. A significant decrease in rosetting was reported with GYPC mAb BRIC 4, although the reduction in the median rosette frequency was small (from 11.5 to 5.5%), and was based on a single count for each isolate with no replication (Lee *et al*., 2014). The biological significance of these results is difficult to assess, given the low starting rosette frequencies and inherent variation in the rosetting assay. Lee *et al.* also used a different definition of rosetting to all previous studies, defining a rosette as an IE binding one or more uninfected erythrocytes. The usual definition requires the binding of two or more uninfected erythrocytes, which helps to identify genuine cell–cell interactions and avoid spurious identification of rosettes due to close packing of cells under the coverslip during microscopy.

For *P. vivax*, Lee *et al.* showed that the GYPC mAb reduced the median rosette frequency from 30 to 22% when tested on 11 Thai isolates, and that GYPC knockdown cultured erythrocytes formed rosettes poorly compared to GYPC-positive control cells (median rosette frequency 6.2 *vs.* 35.4% in controls, tested on three isolates). *Plasmodium falciparum* isolates were not tested with the GYPC knockdown erythrocytes.

If GYPC is a rosetting receptor, it is possible that the ‘Gerbich-negative’ blood group type, which is common in Melanesian populations (Patel *et al*., 2001), might influence rosetting. As part of a screen of null blood group erythrocytes with five high-rosetting *P. falciparum* culture-adapted parasite lines, Rowe *et al*. (1997) tested two donors with the Gerbich-negative blood group (formed by deletion of exon 3 of the *GYPC* gene on chromosome 2, giving a truncated protein with altered glycosylation). Gerbich-negative erythrocytes formed rosettes normally with the five parasite lines tested. Goel *et al.* also report normal rosetting of Gerbich-negative erythrocytes from two donors (Goel *et al*., 2015). The true null phenotype for GYPC, called the Leach phenotype (which arises due to the deletion of exon 3 and exon 4, encoding the transmembrane and cytoplasmic domains, respectively) is rare and has not been tested in rosetting assays to our knowledge.

Taking into account all existing evidence, further investigation of a wider range of parasite lines is needed to determine whether GYPC is an important host receptor for both *P. falciparum* and *P. vivax* rosetting.

### Glycophorin A (GYPA, GPA, CD235a)

GYPA is a highly-expressed erythrocyte surface glycoprotein that carries the MNS blood group antigens. It is known to be a receptor for *P. falciparum* erythrocyte invasion (Sim *et al*., 1994), and polymorphisms in GYPA are associated with resistance to severe malaria (Band *et al*., 2015; Leffler *et al*., 2017).

There is some limited evidence to suggest that GYPA may have a role in rosetting. Parasites of the strain FCR3S1.2 transfected with a specific RIFIN gene formed rosettes that were largely dependent on blood group A (Goel *et al*., 2015). However, rosetting of the RIFIN-transfected parasites was significantly reduced with GYPA null cells from blood group O and B donors, whereas blood group A GYPA null erythrocytes formed rosettes normally. These data suggest that GYPA may have an accessory role for RIFIN-mediated rosetting in the absence of the A antigen (Goel *et al*., 2015), although whether this applies to rosetting in non-genetically manipulated parasites in unknown.

Despite the above positive evidence, there are no other data supporting a role for GYPA in rosetting. GYPA mAb fragments had no inhibitory effect on rosetting in ten *P. falciparum* and 11 *P. vivax* clinical isolates (Lee *et al*., 2014), and a GYPA mAb did not inhibit 3D7 5A-R+ rosettes (Niang *et al*., 2014). Furthermore, GYPA null erythrocytes (MkMk cells, lacking both GYPA and glycophorin B) formed rosettes with five culture-adapted *P. falciparum* lines (Rowe *et al*., 1997). Overall, existing evidence does not support a major role for GYPA in rosetting, but as with GYPC, further investigation is needed.

### New receptors and new approaches

None of the receptors described above fully account for the adhesion interactions between infected and uninfected erythrocytes, and it is likely that other host rosetting receptors remain to be identified. There is evidence to suggest that these unknown host receptors are carbohydrates or protease-resistant proteins, because uninfected group O erythrocytes treated with trypsin and other proteases are still able to form rosettes (Udomsangpetch *et al*., 1989; Rowe *et al*., 1994).

In order to progress rosetting research, alternative methods are needed. Rosetting experiments with GYPC and CR1 knockdown cultured human red cells derived from CD34+ haematopoetic stem cells have been performed (Lee *et al*., 2014; Niang *et al*., 2014), using lentiviral transduction of short hairpin RNA (Bei *et al*., 2010). However, these cultured erythrocytes have a short life-span, limiting their usefulness. The development of immortalized erythroid lines (Kurita *et al*., 2013; Kanjee *et al*., 2017; Trakarnsanga *et al*., 2017; Scully *et al*., 2019) may overcome this limitation. Nevertheless, attention must be paid to the subtle but real differences between mature erythrocytes and these, still relatively immature, immortalized CD34+ derived cells (Wilson *et al*., 2016; Dankwa *et al*., 2017; Trakarnsanga *et al*., 2017). CRISPR-Cas9 technology (Doudna and Charpentier, 2014) has led to an explosion in the ability to genetically manipulate multiple cell types, including erythrocyte precursors and immortalized haematopoietic lines (Song *et al*., 2015; Kanjee *et al*., 2017; Hawksworth *et al*., 2018; Chung *et al*., 2019; Scully *et al*., 2019), potentially giving the opportunity to generate multiple knockout lines for rosetting research. A consistent supply of knockout erythrocytes would allow large-scale screens for new rosetting receptors using cells as close to their normal physiological form as possible, raising exciting prospects for future work.

## Conclusions

Of the rosetting receptors described over the past 30 years, only the blood group A trisaccharide has been authenticated by a variety of methodological approaches from a range of different investigators. For all other potential rosetting receptors, the evidence remains fragmentary (Table 1) and further research is needed (Table 2). Recent technical advances in genetic manipulation of red cell precursors and immortalised lines should enable reverse genetic studies to bring further clarity to this biologically important topic.

**Table 2.** Key areas for future research on rosetting receptors

Determine the relative importance of known host erythrocyte receptors
Develop screening technologies to identify novel host rosetting receptors
Use immortalized erythroid lines for reverse genetic studies in rosetting
Develop novel rosette-disrupting adjunctive therapies
Develop *in vivo* models to test rosette-disrupting adjunctive therapies
